# Development of mental health quality indicators (MHQIs) for inpatient psychiatry based on the interRAI mental health assessment

**DOI:** 10.1186/1472-6963-13-15

**Published:** 2013-01-10

**Authors:** Christopher M Perlman, John P Hirdes, Howard Barbaree, Brant E Fries, Ian McKillop, John N Morris, Terry Rabinowitz

**Affiliations:** 1 School of Public Health and Health Systems, University of Waterloo, 200 University Ave West, Waterloo N2L 3G1, Canada; 2 Waypoint Centre for Mental Healthcare, 500 Church St, Penetanguishene, L9M 1G3, Canada; 3 Institute of Gerontology and School of Medicine, University of Michigan, 300 North Ingalls St, Ann Arbor, 48109, USA; 4 Ann Arbor VA Healthcare Center, 2215 Fuller Rd, Ann Arbor, 48105, USA; 5 Hebrew Senior Life, 1200 Centre Street, Boston, 02131, USA; 6 Fletcher Allen Health Care, 111 Colchester Avenue, Burlington, 05401, USA

**Keywords:** Quality, Performance, Indicators, Outcomes, interRAI, Mental health, Psychiatry, Assessment system

## Abstract

**Background:**

Outcome quality indicators are rarely used to evaluate mental health services because most jurisdictions lack clinical data systems to construct indicators in a meaningful way across mental health providers. As a result, important information about the effectiveness of health services remains unknown. This study examined the feasibility of developing mental health quality indicators (MHQIs) using the Resident Assessment Instrument - Mental Health (RAI-MH), a clinical assessment system mandated for use in Ontario, Canada as well as many other jurisdictions internationally.

**Methods:**

Retrospective analyses were performed on two datasets containing RAI-MH assessments for 1,056 patients from 7 facilities and 34,788 patients from 70 facilities in Ontario, Canada. The RAI-MH was completed by clinical staff of each facility at admission and follow-up, typically at discharge. The RAI-MH includes a breadth of information on symptoms, functioning, socio-demographics, and service utilization. Potential MHQIs were derived by examining the empirical patterns of improvement and incidence in depressive symptoms and cognitive performance across facilities in both sets of data. A prevalence indicator was also constructed to compare restraint use. Logistic regression was used to evaluate risk adjustment of MHQIs using patient case-mix index scores derived from the RAI-MH System for Classification of Inpatient Psychiatry.

**Results:**

Subscales from the RAI-MH, the Depression Severity Index (DSI) and Cognitive Performance Scale (CPS), were found to have good reliability and strong convergent validity. Unadjusted rates of five MHQIs based on the DSI, CPS, and restraints showed substantial variation among facilities in both sets of data. For instance, there was a 29.3% difference between the first and third quartile facility rates of improvement in cognitive performance. The case-mix index score was significantly related to MHQIs for cognitive performance and restraints but had a relatively small impact on adjusted rates/prevalence.

**Conclusions:**

The RAI-MH is a feasible assessment system for deriving MHQIs. Given the breadth of clinical content on the RAI-MH there is an opportunity to expand the number of MHQIs beyond indicators of depression, cognitive performance, and restraints. Further research is needed to improve risk adjustment of the MHQIs for their use in mental health services report card and benchmarking activities.

## Background

Quality indicators support accountability for funding, delivery, effectiveness, and improvement of health services [1,2]. As such, numerous quality indicator initiatives for mental healthcare are evident internationally [3-5] with a large degree of heterogeneity in the types, sophistication, and utility of indicators across countries [6]. Most reporting infrastructures for quality acknowledge the need for structure, process, and outcome indicators [7]. Process indicators are those most commonly used [2,4] and have been the primary focus of benchmarking quality of mental healthcare to date [8,9]. Process indicators consider issues such as safety, accessibility, appropriateness, and timeliness of treatments and services[10]. The use of outcome indicators of mental healthcare, particularly those focused on clinical status, is limited even though there is well documented need [11-13]. Research on mental health outcomes is extensive, but only a handful of studies have used outcome measures to compare quality among providers [3,14-18]. Outcome indicators provide information about the effectiveness of mental health services that, when used in combination with process indicators, can provide information about the link between service delivery and effectiveness [19,20].

The utility of outcome indicators for measuring the quality of mental health services relies on the availability of clinical data sources that are common across providers. In addition to constructing outcome indicators, clinical data are needed for appropriate case mix adjustment [14,15,21-24]. When patient characteristics are strongly related to an outcome of interest and are unequally distributed among providers, comparisons of outcomes may favor providers who have a favorable case mix (i.e., a high number of patients who have “less severe” conditions or illness). Case mix, or risk, adjustment is best applied using person level clinical characteristics [23]; however, other than sociodemographic (e.g., age, sex) and diagnostic information, these data have not been routinely available.

There have been several calls for a common mental health clinical data source that includes patient characteristics, service utilization, and outcomes to support care delivery and quality measurement of inpatient psychiatry [12,25,26]. Responding to similar calls, the Ontario Ministry of Health and Long Term Care (MoHLTC) implemented the Resident Assessment Instrument for Mental Health (RAI-MH; called the interRAI MH internationally) [27,28] for use in all facilities with adult psychiatric hospitals/units beginning in October 2005. The RAI-MH is the primary assessment system used for the Ontario Mental Health Reporting System (OMHRS) of the Canadian Institute for Health Information (CIHI) (http://www.cihi.ca/omhrs). OMHRS produces quarterly reports on patient characteristics, case mix, care needs, outcomes, and quality to all hospitals with mental health beds in Ontario. The interRAI Mental Health has also been implemented in other provinces (e.g., Newfoundland and Labrador, Manitoba) and internationally (e.g., Iceland, Finland). Compatible interRAI systems have been implemented in other health sectors in the United States, Canada, and internationally [29-32].

The RAI-MH is part of a suite of assessment systems available for almost every health sector. The RAI-MH and other interRAI assessments include applications for care planning, case-mix, outcomes, and quality measurement [28]. Most interRAI quality indicator research, to date, has focused on long term care [33-36], home care [37,38], and post-acute care [39]. A set of 35 Mental Health Quality Indicators (MHQIs) was developed based on a previous version of the RAI-MH [40]. The initial MHQIs included outcome and process indicators of emotional, behaviour, and cognitive patterns, nutrition/eating, physical functioning, clinical management, restraints, sexual violence, and accidents. At the time of development, longitudinal data were not available to assess the utility of the initial MHQIs because the MoHLTC had not yet implemented the RAI-MH. When the instrument was implemented, a new version was used that no longer included items to support all domains in the original MHQIs. The current implementation does include data that is collected at multiple points in time during an inpatient stay.

The purpose of this study is to evaluate the feasibility of using the current version of the RAI-MH to derive MHQIs. Choosing several domains from the initial MHQIs, this study will examine RAI-MH content to develop several new MHQIs and explore the utility of these MHQIs for identifying variability across mental health settings. For each MHQI, this study will also examine the utility of a RAI-MH case-mix index variable for risk adjustment. The goal of this study is to introduce the concept and potential utility of the MQHIs based on the RAI-MH for carrying out quality measurement of inpatient mental health services.

## Methods

### Data

Retrospective analyses were performed on two RAI-MH datasets representing inpatients of mental health facilities in Ontario, Canada. The first dataset (pilot data) was used to initially derive several MHQIs. The pilot data included assessments for 1,056 patients from a RAI-MH pilot study in seven volunteer facilities in Ontario conducted between November, 2004 and April, 2005. Clinical staff at participating facilities who received three days of RAI-MH training completed RAI-MH assessments on consecutive admissions of adults aged 18 years and older. For each patient, an admission assessment was completed after the third day of stay and an additional assessment was completed at discharge. The admission assessment was completed after the third day of stay because a number of RAI-MH items are based on 72 hour observation periods. De-identified data were submitted by each facility to the research team at the University of Waterloo. This protocol received approval from the University Of Waterloo Office of Research Ethics and the research ethics boards of participating facilities, where applicable.

The second dataset included 34,788 episodes of care for persons who had two complete RAI-MH assessments submitted to CIHI between October, 2005 and March 31, 2007. Under provincial implementation, the RAI-MH is completed at admission and discharge for each person and submitted quarterly by each facility to CIHI. Data quality checks and reports are completed by the OMHRS team at CIHI and communicated to facilities. These reports include a review of item non-response, data accuracy, coding errors, and other data quality issues (a full overview of data quality can be found at http://www.cihi.ca/omhrs). Assessments with errors are rejected and returned to hospitals for correction. Through a data sharing agreement, CIHI sent a copy of the de-identified data to interRAI through the University of Waterloo.

### Measures

The RAI-MH assesses domains including demographics, mental and physical health symptoms, substance use, behaviors, and service utilization, as well as social, vocational, cognitive, and physical functioning [28,41]. Taking about an hour to complete for an average person, the RAI-MH is completed by clinical staff overseeing the treatment of the person based on interview, observation, and discussion with other members of the care team. The implementation in Ontario hospitals of the RAI-MH was supported by extensive training managed and delivered by CIHI. Each hospital contributing RAI-MH is assigned at least one “RAI Coordinator”, typically a clinical staff member of the hospital who provides ongoing RAI-MH support and training to clinicians. The CIHI also provides ongoing training and support though face to face training sessions, teleconferences, webcasts, and ad hoc email or telephone support. Information from the RAI-MH contributes to the global clinical assessment of the person and is included in clinical documentation. Based on provincial mandate, the completion rate of the RAI-MH in routine care in Ontario is 100% for all persons admitted for at least 72 hours (a different assessment is completed for persons staying less than 72 hours). In reliability studies, the average agreement was 83% for all RAI-MH items [41] and the average weighted Kappa for all RAI-MH items was 0.70 [42], considered by Landis and Koch [43] to be “substantial agreement”.

### Variables used to construct the MHQIs

Three domains were chosen for potential MHQIs: Depressive symptoms, cognitive performance, and restraints. These domains were chosen based on their relevance to rehabilitation of severe mental illness [44] and the emergence of restraint reduction as a leading focus of patient safety in mental health services [45]. A number of sub-scales and items embedded in the RAI-MH were considered for derivation of MHQIs. For depressive symptoms, derivation was based on the Depression Severity Index (DSI). The DSI is a scale that sums responses from the following five items: Sad or pained facial expressions, made negative statements, self-depreciation, expressions of guilt/shame, hopeless. Each item is scored based on observed frequency over the prior 3 days from 0 (not present) to 3 (present daily) creating a total DSI score that ranges from 0 to 15. In pilot testing with 1000 patients from ten Ontario hospitals, the DSI was found to have good internal consistency (Cronbach’s alpha = 0.81) and it was strongly associated with indicators of suicidality (e.g., has a suicide plan)[unpublished data, JPH]. Cognitive Performance was assessed using the Cognitive Performance Scale (CPS). The CPS is based on a decision tree algorithm that includes items assessing daily decision-making, short-term memory, ability to make self-understood, and self-performance in eating. The CPS ranges from 0 to 6 identifying persons with intact (0), borderline intact (1), mild (2), moderate (3), moderate-severe (4), severe (5), or very severe (6) cognitive performance [46]. The CPS has been found to correlate with the Mini-Mental State Examination [47] and the Montreal Cognitive Assessment [48] among persons receiving inpatient mental health services [49]. For restraints, the RAI-MH includes items assessing the number of times in the prior 3-days a person experienced the following restraints: mechanical, chair that prevents rising, and physical/manual restraint by staff. A restraint variable was created and coded as yes (1) if any of these items were coded as present.

### Calculation of MHQIs

One objective of the study was to explore how the items and sub-scales described above could be used to define quality indicators at the facility level for comparison. The initial MHQIs focused on patterns of change in depression and cognitive impairment based on rates of improvement and incidence. Restraint use was assessed based on prevalence in the three days prior to assessment. These indicators were modified for the second generation MHQIs to include failure to improve in the patterns of change for depressive symptoms and cognitive performance. Failure to improve includes any person who is assessed to have symptoms or functional impairment at initial assessment and whose symptoms or impairment does not change by follow-up. This construct is considered an adverse outcome similar to an incidence of symptoms, since the expected trajectory of clinical status over the course of treatment is improvement. Thus, the second generation MHQIs measuring change in clinical status can be defined in two ways: a) improvement and b) incidence or failure to improve. This operationalization is similar to indicators that have been defined and validated for nursing home [36], home care [37,38], and post-acute care sectors [39].

The MHQIs for depressive symptoms and cognitive performance were calculated within facilities using patient level data. For depressive symptoms, the denominator included persons with a score greater than or equal to 3 on the DSI. This threshold was used because scores below 3 would indicate very mild depressive symptoms (less than daily expression of any symptom) that may not be subject to improvement. For cognitive performance, the denominator included persons with scores greater than 0 on the CPS. The numerator for improvement in depressive symptoms or cognitive performance included all persons whose score on the DSI or CPS was lower at follow-up compared to the score at initial assessment. For indicators of incidence or failure to improve, the rate included two groups: 1) Persons who had a score of 0 on either the DSI or CPS at baseline and whose score was greater at follow-up, or 2) Persons with baseline scores above 3 on the DSI or above 0 on the CPS and scores that remained the same or increased at follow-up. The prevalence of restraint use at the time of assessment included all persons with a valid assessment who experienced any one of the three types of restraint in the three days prior to assessment.

### Risk adjustment variable

The RAI-MH System for Classification of Inpatient Psychiatry (SCIPP), a system recently approved for use in funding inpatient psychiatry in Ontario [50], was examined for potential use as a risk adjuster for the MHQIs. Although it was primarily designed to describe resource intensity, this additional use of the SCIPP was inspired by the use of case mix measures for risk adjustment of quality indicators in other health sectors, including long term care [36]. The SCIPP uses RAI-MH items to divide patients into 47 groups based on a hierarchical grouping of provisional diagnosis and other patient characteristics (e.g., behaviours, psychotic symptoms). Each group is assigned a case mix index (SCIPP-CMI) score ranging from 0.26 to 2.17 representing the relative cost of caring for patients. A SCIPP-CMI above 1.00 indicates the patient is more resource intensive than the average patient while SCIPP-CMIs below 1.00 are considered less resource intensive than the average patient. The most resource-intensive group includes patients with schizophrenia, a length of stay less than 3 days, and observed aggressive behavior. The least resource intensive group includes patients with schizophrenia and psychotic or affective symptoms present, lengths of stay of 730 days or more, no indicators of danger to others, and no difficulties in activities of daily living.

### Analysis of MHQIs

Descriptive statistics were calculated for the DSI, CPS, and restraints items to examine the distribution of these scales and items in the OMHRS data. The internal consistency of the DSI was assessed using Chronbach’s alpha. Convergent validity was assessed by examining the bivariate odds of having a mood disorder (DSI) or dementia or other cognitive disorder (CPS) using logistic regression.

The MHQIs were first developed in the pilot data then replicated using the OMHRS data. MHQIs rates were not calculated for facilities with a denominator of less than 20 patients as these rates would be highly unstable [51]. The mean, median, and inter-quartile ranges of MHQI rates among facilities were calculated to describe the distribution of rates between facilities at a single point in time. Each MHQI was reviewed based on reasonable variability (e.g., differences of 10%) in the inter-quartile range between facilities and consistency in rates above 5% and below 95% for all facilities. Variation in MHQI scores among facilities was important for the utility of the MHQI to detect differences in quality. MHQIs with rates consistently below 5% were considered not meaningful for quality monitoring given the infrequency and rarity of the event among all facilities.

The relationship between the SCIPP CMI and each MHQI was examined using logistic regression to evaluate the use of the SCIPP CMI in risk adjustment. The odds ratio and 95% confidence interval was calculated to determine the strength of relationship between the SCIPP CMI. Risk adjustment was then performed for each MHQI in the OMHRS data using the coefficients from the logistic regression. A predicted MHQI score for each observation was calculated using the calculated intercept and SCIPP-CMI regression coefficient, and each observation’s actual SCIPP-CMI score. The facility expected MHQI rate was calculated by averaging the predicted MHQI score of all observations within the facility. Similar to indirect standardization, the risk adjusted MHQI rate was calculated by dividing the facility’s observed MHQI rate by the expected MHQI rate and multiplying by the observed MHQI rate across all facilities. The impact of risk adjustment was assessed by the relationship between facility rankings on unadjusted and adjusted MHQI scores using the Spearman rank order correlation. All analyses were performed using Statistical Analysis Software version 9.1 (SAS, Cary, NC).

## Results

Table 1 shows selected patient characteristics for the pilot and OMHRS data. In the pilot data just over half of patients were under age 45 and had a high school education or less, half were male, and most were without a partner/significant other or spouse. Most patients were considered acute with just under a third having been admitted involuntarily. Common DSM-IV diagnoses included mood disorders and schizophrenia or other psychoses. In the OMHRS data, most patients were between the ages of 25 and 64, about a third were married or had a partner, half had a mood disorder and just over a third had schizophrenia or other psychosis.

**Table 1:** **Characteristics of patients in the pilot and Ontario Mental Health Reporting System** (**OMHRS**) **data**

	**Pilot data**		**OMHRS data**	
**Characteristics**	**N**	**%**	**N**	**%**
**Age categories**				
Under 25	220	21%	4085	12%
25-44	365	35%	14190	41%
45-64	332	31%	11775	34%
65+	139	13%	4725	13%
**Gender(male)**	540	51%	17349	50%
**Marital Status**				
Never Married	404	38%	16563	48%
Married	253	24%	8941	26%
Partner/significant other	56	5%	1126	3%
Widowed	64	6%	1922	6%
Separated	129	12%	2772	8%
Divorced	141	13%	3464	10%
**Education**				
None	129	12%	1304	4
8^th^ grade or less	62	6%	2369	7
9 – 11	181	17%	6856	20
High school	226	21%	8360	24
Technical/trade school	43	4%	1047	3
Some college/university	162	15%	5975	17
Diploma/Bachelor’s degree	105	10%	3688	11
Graduate degree	31	3%	965	3
Unknown	117	11%	4224	12
**Involuntary admission status**	296	28%	8772	25%
**Patient type:**				
Acute	764	72%	27627	79%
Longer term	191	18%	4482	13%
Geriatric	80	8%	1527	4%
Forensic	21	2%	1152	3%
**Provisional DSM**-**IV diagnoses:ˆ**				
Dementia^1^	130	12%	2353	7%
Mood disorder	570	54%	17884	51%
Psychoses^2^	415	39%	13058	37%
Substance-related disorder	225	21%	8634	25%

Note: ˆ indicates that the percentage of persons with provisional psychiatric diagnoses may sum to greater than 100% as persons can have more than one diagnosis. ^1^Delirium, dementia, and amnestic and other cognitive disorders; ^2^Schizophrenia and other psychotic disorders.

In the OMHRS data, the distribution of scores on the, DSI, CPS, and restraint items are found for the total sample and by diagnostic group in Table 2. The mean DSI was 3.9 (SD = 3.8) and the median was 3.0. Scores on the DSI were fairly evenly distributed with 25.6% (n=8891) of persons having a DSI score of 0 and 27.5% (n=9570) scored 6 to 15. Regarding convergent validity, 64% (n=12294) of persons scoring 3 or more on the DSI were found to have a provision diagnosis of mood disorder with the odds of having a mood disorder being 2.12 (95% CI = 2.02, 2.2). The DSI was found to have good internal consistency (Chronbach’s alpha =0.77). The mean CPS score was 0.8 (SD =1.3) and the median 0.0. About 11% (n=3666) of persons scored 3 or higher on the CPS, indicating moderate to very severe impairment in cognitive performance. The prevalence of a delirium, dementia, or other cognitive disorder was 27.4% (n=519) among persons scoring 3 on the CPS (n=1923) and 42% (n=727) where CPS was greater than 3 (n=1743). The odds of a provisional dementia diagnosis was 14.2 (95% CI = 13.0, 15.6) among persons where the CPS was 3 or more. Among all restraints, the most commonly used within three days of admission were mechanical restraints 4.9% (n=1696). Among the four primary diagnostic groups represented in Table 2, the highest prevalence of any restraint use was among persons with dementia (about 14% to 17%).

**Table 2:** **Frequency of the depression severity index**, **cognitive performance scale**, **and restraint items from the RAI**-**MH for the total OMHRS sample and by selected primary provisional DSM**-**IV diagnostic categories**

**RAI**-**MH scales and items used to construct MHQIs**	**Total**		**Primary provisional DSM-IV diagnostic category**							
			**Mood(N**=**14003)**		**Psychoses**^**1**^**(N**=**x11785)**		**Dementia**^**2**^**(N**=**1499)**		**Substance**^**3**^**(N**=**4479)**	
	**Row** %	**N**	**Row** %	**N**	**Row** %	**N**	**Row** %	**N**	**Row** %	**N**
Depression Severity Index (DSI)										
0	25.56	8891	28.1	2496	47.0	4183	5.8	514	12.8	1142
1-2	19.22	6687	35.1	2346	38.5	2574	4.9	331	13.6	912
3-5	27.71	9640	40.3	3884	33.7	3250	4.5	438	12.7	1229
6-12	27.51	9570	55.1	5277	18.6	1778	2.3	216	12.5	1196
Cognitive Performance Scale (CPS)										
0	59.96	20860	44.9	9373	27.8	5798	0.6	122	17.4	3625
1	20.17	7018	37.1	2607	43.7	3064	2.3	159	8.3	583
2	9.33	3244	34.5	1119	44.7	1450	9.2	299	4.6	148
3	5.53	1923	27.4	527	45.4	873	18.1	348	3.9	75
4	0.99	346	22.5	78	47.1	163	17.9	62	2.6	9
5	3.35	1166	21.2	247	33.1	386	34.4	401	3.0	35
6	0.66	231	22.5	52	22.1	51	46.7	108	1.7	4
Restraint Items			**Column %**	**N**	**Column %**	**N**	**Column %**	**N**	**Column %**	**N**
Mechanical restraint	4.9	1696	3.5	491	6.3	740	19.0	285	1.4	61
Chair prevents rising	1.3	414	0.01	75	0.01	66	17.3	259	0.00	6
Physical/manual restraint by staff	3.8	1334	2.7	380	4.8	570	14.7	220	1.4	64

Note: ^1^ Schizophrenia and other psychotic disorders, ^2^Delirium, dementia, and amnestic and other cognitive disorders, ^3^ Substance-related disorders.

Table 3 presents the unadjusted rates and prevalence among the five MHQIs in the Pilot and OMHRS data. In the pilot data, the greatest inter-quartile range among facilities was for the rates of incidence/failure to improve in cognition (34% difference). In the OMHRS data, improvement in cognitive functioning showed the greatest variation (31% difference). Rates of improvement in depressive symptoms tended to be higher in the pilot data compared to OMHRS. Unadjusted prevalence of restraint use also varied substantially in both samples, with facilities in the third quartile having a prevalence of of at least 14.5% in the pilot data and 10.2% in the OMHRS data.

**Table 3:** Unadjusted and adjusted MHQI rates and prevalence among hospitals in the Pilot data and OMHRS data

**Mental Health Quality Indicator**		**PILOT DATA**				**OMHRS DATA**							
		**7 Facilities**				**70 Facilities**							
		**Unadjusted facility MHQI rates**				**Unadjusted facility MHQI rates**				**Adjusted facility MHQI rates**			
**Domain**	**Type of indicator**	**Mean** %	**Median** %	**Q1**	**Q3**	**Mean** %	**Median** %	**Q1**	**Q3**	**Mean** %	**Median** %	**Q1**	**Q3**
Depression	Improvement	86.0	88.6	81.3	89.4	74.0	78.9	69.2	83.9	74.0	78.9	69.2	83.8
	Incidence/Failure to Improve	12.6	10.4	8.5	15.8	25.0	20.8	17.5	30.2	25.0	20.8	17.5	30.2
Cognitive Performance	Improvement	45.7	39.2	37.3	47.9	48.6	49.7	34.7	64.0	48.8	49	34.4	64.1
	Incidence/Failure to Improve	34.0	34.5	16.6	50.7	27.0	25.6	17.1	33.2	26.9	25.3	16.7	33.1
Physical Restraint	Time 1 Prevalence	8.9	4.2	3.4	14.5	7.8	7.3	3.7	10.2	7.6	7.2	3.6	9.9

Logistic regression results examining the association between the SCIPP CMI and unadjusted MHQIs can be found in Table 4. Higher scores on the SCIPP CMI were significantly associated with both the improvement (OR = 1.39, .95CI = 1.29, 1.51) and incidence/failure to improve (OR = 1.23, .95CI = 1.17, 1.31) in cognitive performance. The mean SCIPP CMI was 1.74 (SD = 0.43) among persons who improved in cognitive performance and 1.67 (SD=0.43) among persons who experienced an incidence or failure to improve in cognitive performance. The SCIPP CMI was strongly related to the prevalence of restraint use (OR = 2.71, .95CI = 2.44, 3.01) and was not associated with MHQIs related to change in depressive symptoms.

**Table 4:** **Logistic regression model results for the relationship between the SCIPP CMI and MHQIs based on depressive symptoms**, **cognitive performance**, **and restraint use within three days of admission**

						**95**% **Confidence interval**	
**Mental Health Quality Indicator**		**Coefficient**	**Standard error**	**p**-**value**	**Odds ratio**	**Lower**	**Upper**
Improvement in depressive symptoms	Intercept	1.13	0.06	&0.0001			
	SCIPP_CMI	0.03	0.03	0.37	1.03	0.96	1.10
Incidence/failure to improve in depressive symptoms	Intercept	−1.23	0.05	&0.0001			
	SCIPP_CMI	−0.01	0.03	0.83	0.99	0.94	1.05
Improvement in cognitive performance	Intercept	−0.53	0.07	&0.0001			
	SCIPP_CMI	0.33	0.04	&0.0001	1.39	1.29	1.51
Incidence/failure to improve in cognitive performance	Intercept	−1.53	0.05	&0.0001			
	SCIPP_CMI	0.21	0.03	&0.0001	1.23	1.17	1.31
Prevalence of restraints	Intercept	−4.32	0.10	&0.0001			
	SCIPP_CMI	1.00	0.05	&0.0001	2.71	2.44	3.01

Risk adjustment on the SCIPP CMI had little impact on the MHQI scores among hospitals. Table 3 shows that the mean, median, and inter-quartile range of unadjusted and adjusted MHQIs rates of hospitals in the OMHRS data did not substantially differ. Among MHQIs that were significantly predicted by the SCIPP CMI, adjusted rates were virtually identical across hospitals. The greatest difference in rates was found between unadjusted (interquartile range of 6.5%) and adjusted (interquartile range of 6.3%) prevalence of restraints. Following SCIPP adjustment of the prevalence restraint use, 34% (n=24) of hospital ranks improved, 36% (n=25) declined, and 30% (n=21) stayed the same. However, unadjusted and adjusted ranks on this indicator were highly correlated (r_Spearman_ = 0.99, p&0.0001) indicating that the change in ranks was not substantial. Correlations between ranks on indicators for cognitive performance and depressive symptoms were 1.0 indicating virtually no effect of risk adjustment using the SCIPP CMI.

## Discussion

This study has supported the feasibility of the RAI-MH for developing MHQIs based on three dimensions. However, the findings point to a need for further validation and risk adjustment in order for to support ongoing quality monitoring. The RAI-MH sub-scales used to derive MHQIs for depressive symptoms (the DSI) and cognitive performance (CPS) had acceptable psychometric properties and variability among persons receiving adult inpatient mental health services. Unadjusted rate and prevalence MHQIs derived in this study showed substantial variation among hospitals in both the pilot and OMHRS data and no evidence of ceiling or floor effects were identified. The lower mean rates of improvement for cognitive performance compared to depressive symptoms may be related to the more intensive nature of interventions for cognitive issues (e.g., occupation rehabilitation) compared to faster acting interventions (e.g., pharmaceutical) available for management of depressive symptoms. This finding combined with the variability found among hospitals within each MHQI supports the potential utility of the MHQIs for identifying variations in quality. A lack of variability and consistently high or low rates would indicate that the MHQIs are not responsive to variations in practice between hospitals.

The substantial variance in facility MHQI scores, particularly among indicators measuring changes in cognitive performance, may also be related to the inherent differences in the patient case mix among Ontario hospitals. Evidence was limited for the utility of the SCIPP CMI as a risk adjuster for the five MHQIs explored in this study. Conceptually, the SCIPP CMI would seem to be an appropriate risk adjustment variable since it is a composite measure of diagnosis and patient characteristics that are organized to produce scores reflecting greater resource intensity. Since it is used for reimbursement, adjusting based on the SCIPP CMI would have potential to protect against inappropriately rewarding or penalizing facilities for treating either higher or lower risk patients. Although significant relationships were found between the SCIPP CMI and MHQIs for cognitive performance and restraints, adjustment using the SCIPP CMI led to virtually no difference between unadjusted and adjusted hospital MHQI rates or prevalence. The lack of effect in risk adjustment may be related to the composition of the SCIPP CMI. It also suggests that different MHQIs are likely to require different risk adjusters and that at least some of these will require more specific, rather than global, measures of risk. In addition, although SCIPP includes variables that would be expected to affect risk adjustment (e.g., diagnosis) the variables that largely drive resource intensity (e.g., behaviours, suicidality, day of stay, positive symptoms) are also those that are likely to improve rapidly once treatment is initiated. Thus, while they are related to greater resource utilization at a point in time they may not be related to the likelihood of good or poor outcome over time. Instead, case mix adjustment of MHQIs should focus on the use of singular clinical constructs (e.g., items or symptom scales) that impede clinical efficacy. The broad set of clinical information assessed by the RAI-MH will be useful for improving the effectiveness of risk adjustment of MHQIs. Further research is needed to identify case mix adjusters of MHQIs using variables representing singular clinical constructs (e.g., items or symptom scales) rather than composite indices of resource intensity.

In addition to case mix differences, the variability in MHQI scores may also reflect differences in care processes between hospitals. Detecting such differences is an important attribute for quality indicators as it links the measurement of quality to specific opportunities for quality improvement. The variability in preliminary MHQI findings between Ontario hospitals could be related to differences in measurement properties, ascertainment, policy, or practice. Further analysis of the sensitively to change of the RAI-MH scales are needed to determine if variability in the MHQIs is attributable to the sensitivity to change of the scales or actual variations quality. Regarding ascertainment, a thorough training program for completing the RAI-MH is provided to hospital clinicians by CIHI, “refresher” training may ensure clinical staff are able to appropriately ascertain clinical dimensions of these and future MHQIs. Ongoing data quality monitoring should examine inter-rater reliability in periodic sub-sample evaluations to identify opportunitiesion for the RAI-MH. Practice issues may be explored through a comparison between the MHQIs and other established quality indicators. Once risk adjustment is improved, establishing a relationship between the MHQIs and technical process indicators (e.g., staffing levels, use of evidence based practices, availability of ongoing assessment training, etc.) may identify other contextual factors related to differences in MHQI scores between facilities. More importantly, linking the quality indicators to specific care planning activities may be more effective in understanding how care processes relate to outcomes. The RAI-MH includes care planning applications called Clinical Assessment Protocols (CAPs; formerly Mental Health Assessment Protocols) [52,53] that identify patient problems or needs to staff and include a series of guidelines that staff can use to intervene if deemed necessary. Several CAPs may relate to potential domains of outcome and quality including aggressive behavior and violence, financial and medication management, activities of daily living, pain, interpersonal conflict, acute control medication and physical restraint use. Examining the relationship between use of the CAPs and outcomes based on the MHQI will be important to further validate the MHQIs and provide a mechanism for engaging clinical staff in the quality measurement and improvement process directly at the point of care.

The methodology used in this study could be applied to derivation of MHQIs based on other domains of quality and outcome. In addition to depressive symptoms, cognitive performance, and restraints, the RAI-MH includes a number of other domains of symptoms, behaviours, functioning, and safety. For instance, the RAI-MH includes a positive symptoms scale, pain scale [54], aggressive behavior scale [55], activities of daily living scales [56], and items measuring social functioning, violent and disruptive behavior, financial and medication management, and chemical restraint use are also available [28,41]. Given the feasibility of the RAI-MH for designing quality indicators, these dimensions should be explored for the development of an inventory of RAI-MH MHQIs.

Several limitations of this study should be noted. First, the pilot data were drawn from only seven facilities that used the RAI-MH prior to the provincial mandate; however, the availability of OMHRS data made it possible to replicate the findings guiding initial MHQI selection. Second, the MHQIs were derived from data that excluded patients with stays of less than 6 days and had only one assessment available. Establishing outcome MHQIs for these may be limited to prevalence indicators of events such as self harm, harm to others, and control procedures. Third, several potential MHQIs measuring the appropriateness of medication use were excluded because medication data was unavailable among all facilities. While all interRAI instruments include sections on medication use, the OMHRS data requirements do not include mandated submission of medication data. Given the importance of pharmaceutical therapies as part of psychiatric services, the lack of these data is an important limitation of the OMHRS database. Without medication data inferences cannot be made about differences in quality between facilities that could be related to the appropriate use of medications.

The current dichotomy used in the MHQIs to measure change does not account for the magnitude of change in a given domain. Indicators assessing magnitude of change would complement the MHQIs by providing information on the clinical efficiency of mental health services in addition to effectiveness. Research is currently underway by the authors to examine the sensitivity to change of the RAI-MH sub-scales, including the DSI and CPS, in order to develop indicators measuring clinical efficiency based on the magnitude of change in the MHQI domains over time. In combination, the MHQIs and efficiency indicators could improve the accuracy of facility rankings by combining information on the proportion of patients who change with the magnitude of that change.

The MHQIs, once validated, carry several advantages for quality improvement at the person and organization levels. The MHQIs examined in this study measure domains that are important for the person’s recovery process and safety. Supporting improvements in cognitive functioning, for instance, may help the person achieve and sustain integration back into community settings. The measurement of such outcomes in accountability frameworks and report cards reinforces interventions directed at improvement, thus benefiting patients with such needs. At the organizational level, the inclusion of improvement as well as incidence/failure to improve in many MHQI domains emphasizes success and opportunities for improvement. The use of prevalence indicators for restraints emphasizes patient safety. Combined, these dimensions will be useful for supporting quality improvement by identifying and sharing best practices between higher and lower ranking facilities based on theses and, potentially, other MHQIs.

Once further refinement is complete, the MHQIs may also be applicable for quality measurement across health sectors, from inpatient to community mental health and beyond. All interRAI instruments include core items that are consistent across all assessments as well as items that are specific to the sector being measured [42]. The interRAI Community Mental Health (CMH), for instance, contains 60% of the items used in the RAI-MH. Only the restraints MHQI cannot be measured using the interRAI CMH. Completion of the interRAI CMH 30 days after discharge, for instance, could serve as a third follow-up assessment for inpatient MHQIs and a baseline assessment for community MHQIs. The interRAI CMH has been pilot tested in Canada (Ontario, Newfoundland), Iceland, Cuba, and Chile, but it has not yet been mandated for regular use.

## Conclusions

This study has provided support for the utility of information from the RAI-MH for measuring and comparing quality indicators of process and outcome between facilities. Detailed refinement is needed to fully establish the reliability and validity of MHQIs based on the RAI-MH. Prior to this refinement, conclusions about quality care based on the indicators in their current state may not be appropriate. Further progress in establishing valid and reliable MHQIs based on the RAI-MH may identify outcome indicators that can be used for a number of important psychiatric domains, including symptom, functional, behavioral, and safety dimensions. This refinement should include additional risk adjustment strategies for the MHQIs to support their use. Once further refinement is complete, opportunities exist to use the MHQIs for identifying quality improvement opportunities and comparing quality regionally, nationally, and internationally, wherever the RAI-MH is used.

## Abbreviations

CAP: Clinical Assessment Protocol; CIHI: Canadian Institute for Health Information; CMI: Case Mix Index; CPS: Cognitive Performance Scale; DSI: Depression Severity Index; interRAI CMH: InterRAI Community Mental Health; MHQI: Mental Health Quality Indicator; MoHLTC: Ministry of Health and Long Term Care; OMHRS: Ontario Mental Health Reporting System; SCIPP: System for Classification of Inpatient Psychiatry; RAI-MH: Resident Assessment Instrument Mental Health.

## Competing interests

The authors declare that they have no competing interests.

## Authors’ contributions

CP participated in the study design, performed the statistical analyses and drafted of the manuscript. JPH conceived the study, participated in the study design and statistical analyses, and helped to draft the manuscript. HB, BF, IM, JNM, and TR participated in study design, contributed to the interpretation of the statistical analyses, and helped to draft the manuscript. All authors read and approved the final manuscript.

## Pre-publication history

The pre-publication history for this paper can be accessed here:

http://www.biomedcentral.com/1472-6963/13/15/prepub
